# Stocky/Packed Pancreas: A Case of Focal Drug-Induced Acute Pancreatitis Mimicking Cancer

**DOI:** 10.3390/tomography8040174

**Published:** 2022-08-19

**Authors:** Marco Di Serafino, Roberto Ronza, Divina D’Auria, Roberto Fiorentino, Dario Arundine, Annalisa De Leone, Salvatore Picascia, Alberto Martino, Enrico Crolla, Severo Campione, Giovanna Guida, Carlo Molino, Ferdinando Riccardi, Luigia Romano

**Affiliations:** 1 Department of General and Emergency Radiology, “Antonio Cardarelli” Hospital, 80131 Naples, Italy; 2 Multidisciplinary Team for Pancreatic Cancer, “Antonio Cardarelli” Hospital, 80131 Naples, Italy; 3 Department of Advanced Biomedical Sciences, University of Naples “Federico II”, 80131 Naples, Italy; 4 Department of Oncology, “Antonio Cardarelli” Hospital, 80131 Naples, Italy; 5 Department of Gastroenterology and Digestive Endoscopy, “Antonio Cardarelli” Hospital, 80131 Naples, Italy; 6 Department of Oncological Surgery Team 1, “Antonio Cardarelli” Hospital, 80131 Naples, Italy; 7 Department of Pathology, “Antonio Cardarelli” Hospital, 80131 Naples, Italy; 8 Department of Radiation Oncology, National Cancer Institute–IRCCS–Fondazione G. Pascale, 80131 Naples, Italy

**Keywords:** drug-induced pancreatitis, pancreatic cancer, auto immune pancreatitis, IgG4-related sclerosing disease, FDG PET/CT, MRI

## Abstract

Drug-induced acute pancreatitis (DIP) is a recognised but underreported entity in the literature. Immunotherapy drugs have been described as one possible emerging cause, although the pathogenic mechanism is still largely unclear. To date, only a few cases have been reported, even if in recent times there is an over-increasing awareness of this pathologic entity. The imaging-based diagnosis of DIP can be difficult to establish, representing a real challenge for a radiologist, especially when the inflammatory disease appears as a focal mass suspicious for a malignancy. Case report: We herein report the case of a 71-year-old man with a known history of partially responsive lung adenocarcinoma subtype with high programmed cell death ligand 1 (PD-L1) expression, who underwent positron emission tomography (PET)/computed tomography (CT) imaging follow-up after one year of immunotherapy. The exam revealed a stocky/packed lesion in the pancreatic body, with increased ^18^F-fluorodeoxyglucose (FDG) accumulation highly suggestive of pancreatic cancer, which finally was proven to be a DIP induced by immunotherapy. Conclusion: Distinguishing between focal DIP and pancreatic neoplasm is, therefore, crucial for timely therapeutic management and prognostic stratification. A deep knowledge of possible imaging pitfalls coupled with a comprehensive clinical and laboratory assessment is pivotal to avoid any delays in diagnosis.

## 1. Background

Acute pancreatitis (AP) is an inflammatory disorder of the pancreas characterised by oedema, haemorrhage and necrosis due to pancreatic enzymes autodigestion. Inflammation can be caused by several different aetiologies [1]. Long-standing alcohol consumption and biliary stone disease are considered the most common precipitating factors for AP, but numerous other etiologies are known with increasing evidence that medications can also cause this condition. Although rare, these may lead to the development of acute drug-induced pancreatitis (DIP) [2]. In recent years, many medications have been linked to DIP [3], including chemotherapy and immunotherapy [1,4].

Among the differential diagnoses, primary and secondary pancreatic neoplasm may represent a possible diagnostic pitfall due to the somewhat similar presentation on imaging of acute focal DIP. Thus, a timely differentiation between focal DIP and pancreatic cancer is imperative because of their divergent clinical course and therapeutic management [5]. Here we present a case of acute DIP presenting as a focal pancreatic mass in a patient with primitive lung cancer under immunotherapy. This case illustrates the importance of familiarity with DIP clinical and imaging presentation to optimise patients’ management and avoid any possible diagnostic delay that could affect the final prognosis.

## 2. Case Report

We report the case of a 71-year-old man with a known history of lung adenocarcinoma subtype with high programmed cell death ligand-1(PD-L1) expression, who was referred to our hospital for a scheduled evaluation. The patient was initially diagnosed with inoperable metastatic cancer with large supra-clavear nodal lesions; therefore, he had been under immunotherapy for one year (pembrolizumab).

**Figure 1:**
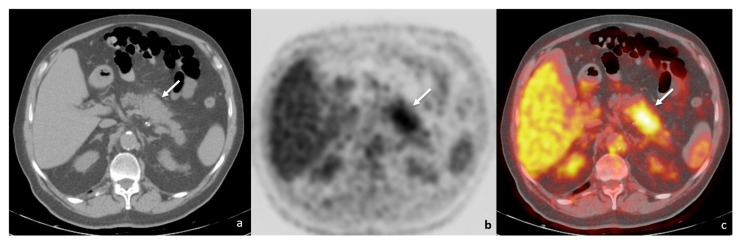
PET/CT ((**a**)—unenhanced CT; (**b**)—positron emission tomography; (**c**)—fusion imaging) showing a tumefactive alteration of the pancreatic body-tail (white arrows), with increased FDG accumulation (SUV = 4.4).

At one year follow-up, positron emission tomography/computed tomography imaging (PET/CT) showed a mass-forming lesion in the pancreatic body and tail, with increased ^18^F-fluorodeoxyglucose (FDG) uptake (maximum standardised uptake value, SUV: 4.4) (Figure 1); no other FDG-avid lesion was present in adjacent lymph nodes, either in the retroperitoneum or in other abdominal parenchyma. The underlying neoplastic disease showed a good response rate, with almost complete regression of both the primitive lung lesion and the secondary node localisations.

From a clinical point of view, at examination, the patient only reported mild upper abdominal pain in the last few weeks. Subsequent routine blood chemistry showed elevated serum pancreatic amylase isoenzyme (90 UI/L; n.v. < 53UI/L), lipase (251 UI/L; n.v. < 60 UI/L) and C-reactive protein (26.7 mg/L; n.v. < 5 mg/L); tumour markers (CEA, CA19-9, CA125, CA15.3) were unremarkable. His previous medical history was negative for long-term course chronic pancreatitis.

Due to the presence of a PET/CT hypermetabolic pancreatic mass not allowing for differential diagnosis between DIP and pancreatic cancer, the patient underwent further examinations, including contrast-enhanced CT and upper abdomen contrast-enhanced magnetic resonance imaging (MRI).

**Figure 2:**
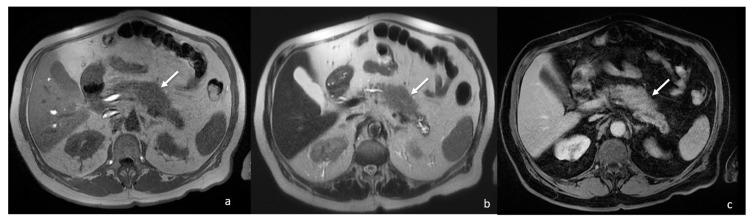
MRI showing focal enlargement of pancreatic body (white arrows) iso-intense on T1w (**a**) and iso-hyper intense on T2w (**b**), with homogeneous enhancement after gadolinium-based contrast agent administration (**c**).

The contrast-enhanced abdomen CT scan showed a 4 cm focal enlargement of the pancreatic body and tail with local narrowing of the main pancreatic duct and abnormal upstream dilatation. The main vessels were not infiltrated, and the intrahepatic and extrahepatic bile ducts were regular. Due to the need to rule out a malignant pancreatic tumour, a contrast-enhanced MRI was then also performed. The MRI confirmed the presence of a stocky/packed poorly defined mass in the pancreatic body and tail, iso- and hypointense on T1w and hyperintense on T2w, with moderate post-contrast enhancement (Figure 2).

**Figure 3:**
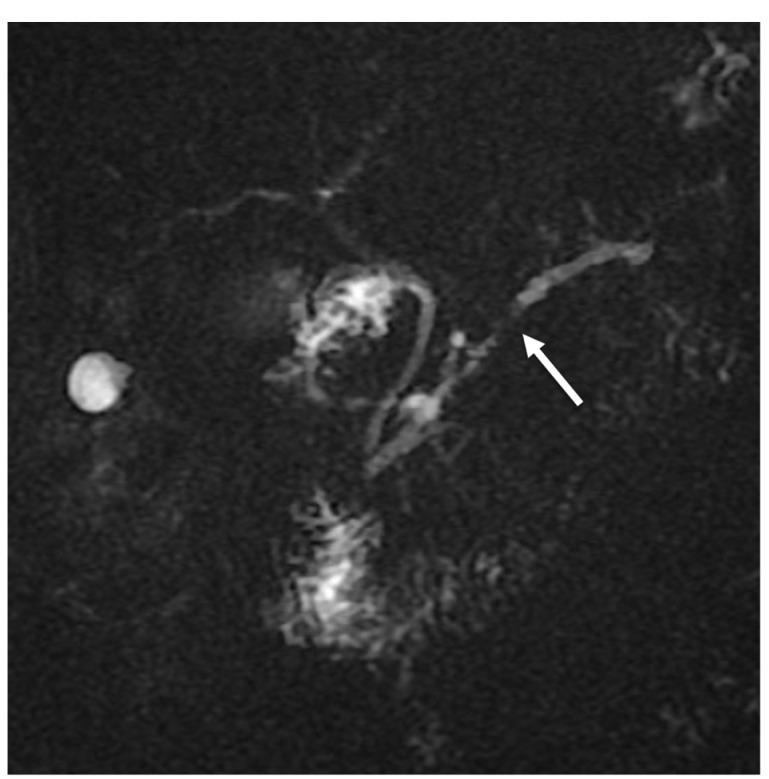
MRCP showing focal main pancreatic duct caliber reduction at the level of the lesion with a slight dilatation above (white arrow).

The mass caused a focal thinning in the main pancreatic duct, which was still visible and free from obstruction, as better shown by cholangiopancreatography (MRCP) (Figure 3) and more suggestive for pancreatitis than pancreatic carcinoma. Furthermore, an MRI showed no sign of vascular infiltration.

**Figure 4:**
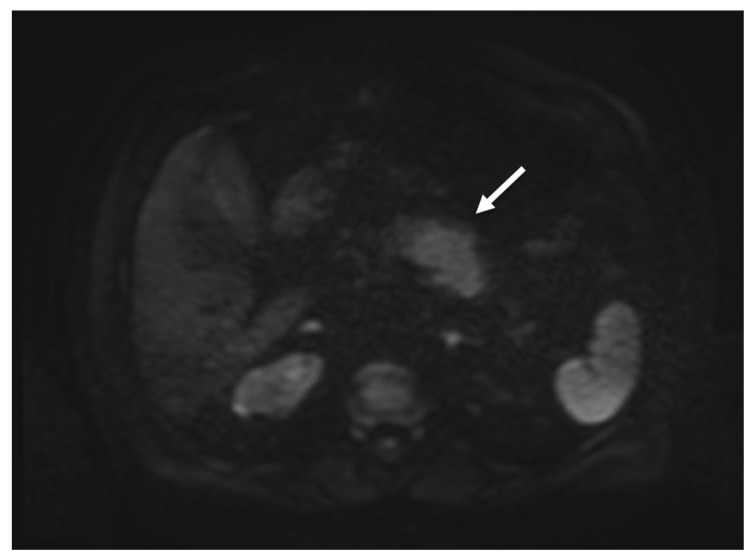
MRI showing focal enlargement of pancreatic body with restricted water diffusion on DWI (b value = 800) (white arrow).

On diffusion-weighted imaging (DWI, b-value = 0-400-800) and relative apparent diffusion coefficient (ADC) maps, the lesion showed inhomogeneous diffusion restriction (mean value about 1000 * 10^−6^ mm^2^/s), which is not highly consistent with the suspicion of pancreatic cancer (Figure 4).

**Figure 5:**
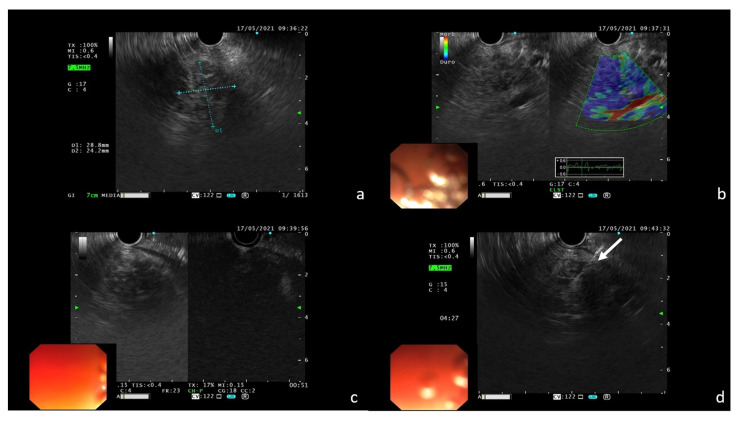
EUS features of the pancreatic lesion confirming the presence of a nodular area in the pancreas body (**a**); dominant blue color at elastography is an indicator of high tissue stiffness (**b**); poor contrast enhancement after i.v. administration of SonoVue (**c**); FNA with 22 G needle of the nodular area ((**d**); white arrow).

Finally, echo-endoscopic ultrasound (EUS)-guided fine needle aspiration (FNA) with a 22 G needle was performed to confirm the suspicion of pembrolizumab-induced DIP. During EUS, the nodular area in the pancreas body to be biopsied showed high tissue stiffness at the elastography mode and poor contrast enhancement after intravenous (i.v.) administration of the ultrasound contrast agent (SonoVue, BRACCO^(C)^ Milan, Italy) (Figure 5).

**Figure 6:**
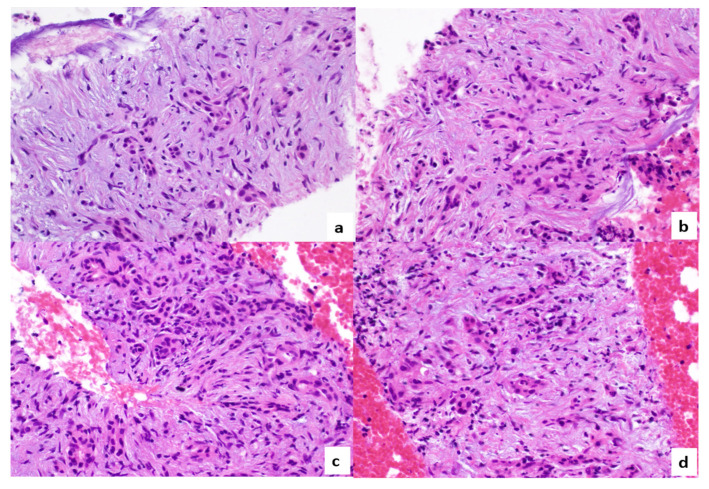
Histopathological specimen ((**a**–**d**); hematoxylin eosin; 400× magnification): pancreatic exocrine tissue presented with marked atrophy, residual acini are distorted, and some inflammatory cells are identifiable.

Subsequent pathologic examination revealed the presence of pancreatic exocrine tissue with marked atrophy, inflammatory infiltration, and some distorted residual acini; no malignant cells were observed within the tissue sample (Figure 6).

**Figure 7:**
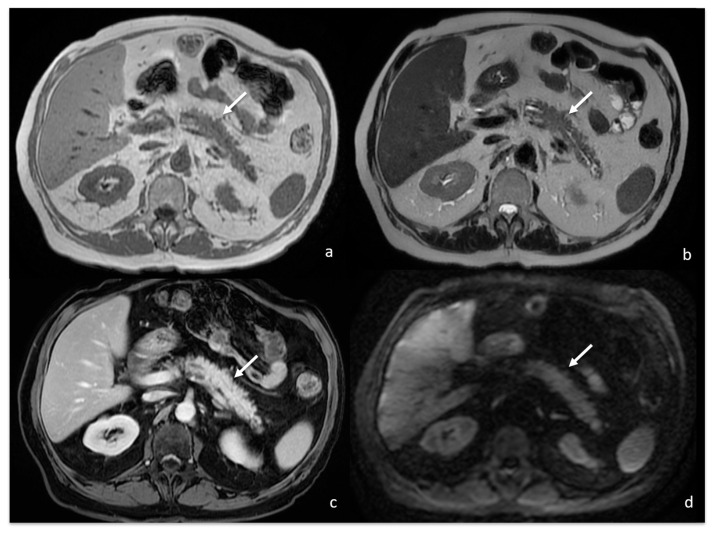
MRI follow-up 1 month after previous examination showing reduction in the previously described focal enlargement of pancreatic body (white arrows) isointense on T1w (**a**) and T2w (**b**), with homogeneous enhancement after gadolinium-based contrast agent administration (**c**) and no restricted water diffusion on DWI ((**d**); b value = 800).

**Figure 8:**
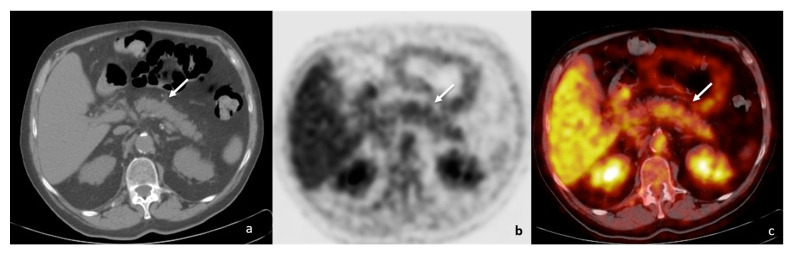
PET/CT ((**a**)—unenhanced CT; (**b**)—positron emission tomography; (**c**)—fusion imaging) at 2 months follow-up confirmed radiological resolution as well as absence of pathologic FDG uptake (white arrows).

The patient was finally diagnosed with focal DIP. Pembrolizumab was discontinued and systemic corticosteroids were administered. A two-month radiological follow-up showed complete DIP regression without pancreatic mass in an MRI (Figure 7) and PET/CT examinations, with an absence of pathologic FDG uptake too (Figure 8). Moreover, no further DIP relapse was observed during longitudinal follow-up.

## 3. Discussion

DIP is a well-documented, although relatively rare, medical condition, in which pancreatitis occurs during treatment with a specific drug, resolves upon discontinuation of the putative agent, and recurs in case of re-administration of the same drug, given that other possible causes of pancreatitis are not present or have been excluded [6]. The first cases of DIP were reported with chlortalidone and cortisone in the 1950s [7]. Since then, over 200 medications have been associated with pancreatitis, including diuretics, oestrogens, and angiotensin-converting enzyme inhibitors [8]. However, due to the low incidence and the lack of pathognomonic clinical hallmarks, determining the true incidence of DIP remains very challenging. Similarly, the pathogenic mechanisms and associated risk factors are mostly unknown. Drugs are overall responsible for an estimated 0.1%–2% of AP incidence; however, the actual prevalence of DIP is thought to be underestimated [9].

According to the recent literature, immunotherapy drugs have been called into question as a possible cause of DIPs [1,2], as observed in the presented case. Among them, immune checkpoint inhibitors (ICIs) are a class of new-generation immunotherapy drugs, currently available for the treatment of different types of cancer, including lung carcinoma [10,11]. These drugs are divided into the following subgroups according to their molecular target: programmed cell death inhibitors-1 (PD-1), programmed cell death ligand-1 (PD-L1), and cytotoxic associated T-lymphocytic molecule (CTLA-4). The activation of the immune cascade against neoplastic tissue mediated by ICIs may also imply a marginal but non-negligible effect on healthy tissues within the body. Such effects, also named adverse events related to the immune system (IrAEs), occur through a mechanism not yet fully elucidated [3,12,13]. With the increased use of ICI, side effects have become more frequent [11]. Among the possible effects, DIPs represent a potentially severe or even fatal complication. Therefore, prompt identification is desirable to optimise patients’ management and avoid negative prognostic repercussions (an issue even more relevant in the case of neoplastic patients) [3].

The reported case represents an example of the challenging differential diagnosis between DIP, other causes of pancreas inflammation, and pancreatic cancer, due to both the relatively unusual disease presentation and the past medical history of the patient that could represent an additive confounder. In our case, DIP manifested as a focal mass conferring to the pancreatic body and tail a characteristic “stocky or packed” appearance, thus potentially mimicking a local malignancy. The differential diagnoses considered should, therefore, always include mass-forming chronic pancreatitis (MFCP) or focal pancreatitis (FP), focal autoimmune pancreatitis (f-AIP), and pancreatic cancer.

MFCP and f-AIP are confined pancreatic inflammations that may both cause pancreas tumefaction; in these cases, clinical history, laboratory testing, and pharmacological anamnesis are pivotal for differential diagnosis. In particular, MFCP develops only during the long-term course of chronic pancreatitis, with the presence of fibrosis as a result of acute inflammation during relapsing pancreatitis. Conversely, f-AIP can be recognised by increased serum IgG4 levels mediated by autoimmune mechanisms. From a radiological point of view, imaging features of f-AIP include (1) delayed homogeneous enhancement, (2) hypoattenuating capsule-like rim, (3) the absence of distal pancreatic atrophy, (4) irregular narrowing of the main pancreatic duct, and (5) stenosis of the common bile duct in patients with lesions in the body or tail [14,15,16]. In our case, MFCP and f-AIP were excluded due to the patient’s silent previous medical history for chronic pancreatitis, the absence of high IgG4 levels at blood chemistry, and, probably most importantly, the lack of characteristic imaging findings.

**Figure 9:**
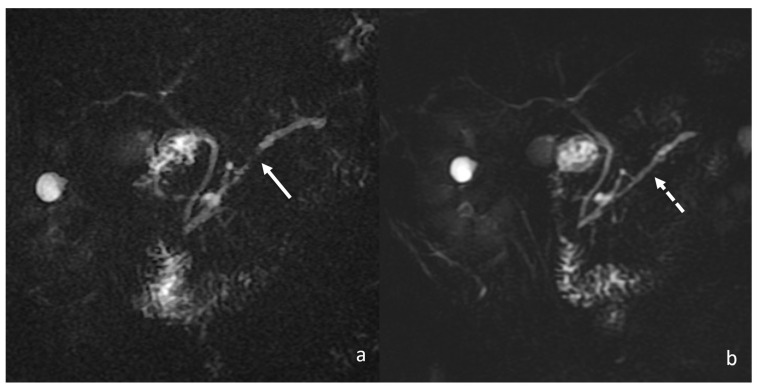
Comparison between magnetic resonance cholangiopancreatography (MRCP) findings at the diagnosis (**a**) and at 1 month follow-up (**b**); focal main pancreatic duct caliber reduction at the level of the lesion with a slight dilatation above (white arrow) completely regressed at 1 month follow-up MRCP (dotted white arrow).

Another important differential diagnosis of DIP encompasses all the possible forms of pancreatic neoplasm, both primary and secondary. Accurate anamnestic collection is the first step to addressing the most appropriate diagnosis. However, as in the presented case, a past medical history of pancreatic or systemic cancer may represent a possible confounder. In a limited number of cases, the evaluation of laboratory tests and multimodal imaging acquisition represent a major turning point in determining the correct diagnosis. Tumour markers have been evaluated to differentiate benign from malignant pancreatic masses. However, such markers, including CA19-9, lack sensitivity for early or small-diameter pancreatic cancer; therefore, they cannot accurately differentiate inflammatory lesions from cancer. For this purpose, an MRI and a PET/CT were performed because they are the most relevant imaging weapons in guiding physicians [17]. The main MRI characteristics include hypointense signal on both T1w and T2w sequences, with slower post-contrast enhancement than the normal pancreas; therefore, dynamic contrast-enhanced sequences with fat saturation are always included. MRCP is also performed to better evaluate the intra- and extra-hepatic biliary tree and pancreatic ductal system focusing on ductal strictures secondary to pancreatic mass (Figure 9). Peripancreatic lymphadenopathy and vascular invasion can also be assessed, although they are not always of univocal interpretation [16].

Given all the reported findings, the diagnosis of focal DIP can be considered a combination of radiological, clinical, and biochemical abnormalities [18,19]. From a laboratory point of view, the lack of neoplastic markers and the presence of specific signs of pancreas flogosis in the blood chemistry suggested the possibility of DIP. On imaging, the association of different modalities may corroborate the diagnostic suspicion of DIP. Although the PET/CT increase in FDG uptake within the pancreatic mass-forming lesion is common to pancreatic cancer and inflammation, it has been demonstrated that SUV values have different benchmarks in the two conditions. Several studies have suggested that the FDG SUV of a lesion was usually greater than 4.0 in patients with pancreatic cancer, 3.0–4.0 in chronic pancreatitis patients, and below 3.0 in healthy volunteers [14]. In patients with AIP, PET/CT showed an intense uptake of FDG by the pancreas, including cases of diffuse and localised uptake. The median SUVmax was 5.0. Therefore, the overall values of FDG uptake by the pancreas did not differ significantly between AIP and pancreatic cancer. PET/CT has shown similar sensitivity and higher specificity compared to contrast-enhanced CT and contrast-enhanced MRI, helping to reduce the rate of incorrect diagnoses more than simple morphological images, especially when combined with the dosage of tumour markers such as CA19-9 [20].

Despite this consideration, functional PET/CT imaging should always be coupled with morphological studies to confirm the suspicion and look for further findings supporting the diagnostic suspicion of pancreatic cancer vs. DIP. During a CT scan, DIPs appear as hypoattenuating masses with an unenhanced CT, poorly vascular after contrast media administration; the lesions are most often located in the pancreatic head. The body or tail of the pancreas can also be involved. Arterial and portal CT phases can help in assessing vascular infiltration, which is suggestive of pancreatic cancer rather than DIP. Similar findings can also be observed in an MRI. However, an MRI has the potential to better assess any potential alteration of the pancreatic duct thanks to the use of dedicated sequences such as MRCP. As shown in the case, the presence of a smooth narrowing of the pancreatic duct traversing the pancreatic mass without abrupt or complete obstruction is highly evocative of inflammatory disorders rather than infiltrative lesions. An inflammatory mass often results in gradual stenosis with residual visualisation of the stenotic pancreatic duct throughout the mass, whereas in the case of a tumour, besides the upstream dilatation, the duct presents with characteristic contour irregularities due to wall invasion, a finding that is not visible in inflammatory pseudo-tumours [17,21]. Similar to a CT scan, an MRI can provide useful information concerning vessel encasement and perivascular oedema. Concerning the signal intensity on MRI examination, both tumours and pseudo-tumours are of an ill-defined mass iso- to hypo-intense on fat-suppressed T1w and on pancreatic parenchymal phase, dynamically enhanced, fat-suppressed, T1w and a variable appearance on T2w, which is often hyper-intense [22]. The use of DWI and relative ADC measurements can represent the most useful tool for detecting focal DIP and distinguishing inflammation with high sensitivity and specificity [23]. Focal DIP is characterised by restricted water diffusion on DWI compared to the surrounding normal pancreatic gland, corresponding to low values on ADC maps. Similarly, neoplastic lesions show true restricted water diffusion due to increased cellularity, also resulting in low ADC values [24]. Despite the similar appearance on DWI, several authors have observed lower ADC values for AIP and DIP compared to pancreatic cancer, probably reflecting the capillary bed recruitment and the increased capillary permeability mediating the over-migration of inflammatory cells such as lymphocytes and macrophages. However, at present, a specific ADC cut-off or benchmark has not been identified because DWI is largely influenced by magnetic field strength, scanner vendor, sequence type, acquisition parameters, and so on. Therefore, each institute should optimise DWI protocols according to system possibilities. In particular, it must be kept in mind that, among the potentially influential parameters, a central role is played by the b value, which is recommended to be at least equal to 600 s/mm^2^ to ensure an optimal signal-to-noise ratio [22,25,26].

Finally, despite multimodal morphological and functional imaging being able to usually guide the correct diagnosis, in doubtful cases or when timely therapeutic intervention is required to avoid potential negative prognostic implications, a direct biopsy (generally EUS- guided) may be envisaged. Once DIP diagnosis is confirmed, any potentially causative drugs should be discontinued or replaced and, more importantly, never administered again due to the risk of relapses [3]. When biopsy can be procrastinated and is not imperative due to the exclusion of possible mimics other than DIP, an ex juvantibus diagnosis can be performed by discontinuation of the suspected putative drug and strict follow-up with the patient, confirming the regression of the pancreatic inflammation with complete ad integrum restitution.

## 4. Conclusions

Various inflammatory abnormalities of the pancreas can mimic pancreatic cancer, including focal DIP. Thus, differentiation between this benign condition and pancreatic cancer is crucial to avoid unnecessary surgery and not delay surgery in case of malignancy. With the presented case, we aimed to highlight the importance of clinical data collection coupled with multimodal imaging assessment for ensuring the most appropriate and timely patient management. Despite the central role of nuclear medicine investigation in distinguishing between mass-forming pancreatitis and pancreatic cancer, morphological studies are warranted to confirm the suspicion, look for further supporting findings, and non-invasively monitor the disease evolution over time. However, in selected patients or cases of high diagnostic uncertainty, a biopsy may be required for histological confirmation.

## Data Availability

Data sharing is not applicable.
